# Revision anterior cruciate ligament reconstruction and additional surgeries: A review

**DOI:** 10.1097/MD.0000000000042620

**Published:** 2025-05-23

**Authors:** Tian-Wang Zhu, Rui-Xin Li

**Affiliations:** a Department of Sports Medicine, Dalian University Affiliated Xinhua Hospital, Dalian, Liaoning, China.

**Keywords:** 1-stage revision anterior cruciate ligament reconstruction (ACLR), bone allograft, bone autograft, bone grafting, bone tunnel widening, extra-articular augmentation, over-the-top technique

## Abstract

Anterior cruciate ligament (ACL) failure is caused by medical and nonmedical factors. A thorough preoperative evaluation should include knowledge of previous injuries, physical examination, and imaging. Classic tunnel management techniques include divergent drilling technique, transtibial technique, anteromedial portal technique, outside-in technique, and over-the-top technique. Although critical tunnel widening or overlap usually requires 2-stage revision ACL reconstruction (ACLR), efforts have been made to perform 1-stage revision ACLR in these cases. Bone grafts include bone autografts and bone allografts, synthetic bone grafts, and biologics. Grafts include autografts, allografts, and artificial ligaments. Extra-articular augmentation benefits revision ACLR in selected individuals. Varus knee and excessive posterior tibial slope should be addressed in some cases. Meniscus injury and articular cartilage injury should be addressed. Although the overall outcomes of revision ACLR are worse than those of primary ACLR, revision ACLR remains important for improving knee function and return to sport. Future research should expand the indications for 1-stage revision, clarify the indications of anterior closing wedge high tibial osteotomy and extra-articular augmentation at the time of revision ACLR, based on strong evidence.

## 1. Introduction

Anterior cruciate ligament (ACL) ruptures are among the most common sports injuries and cause significant economic costs and long-term work absences.^[1]^ Although ACL reconstruction (ACLR) is widely performed, ACLR failure remains a significant and concerning outcome. For patients with ACLR failure, revision ACLR is necessary to restore knee function and quality of life. However, bone tunnel widening and overlap, less available autografts, and more prevalent meniscus and articular cartilage injuries at the time of revision ACLR, compared with the primary ACLR, make revision ACLR particularly complicated and challenging.^[2]^ Additionally, the indications for extra-articular augmentation at the time of revision ACLR remain unclear. This article reviews the etiology of ACLR failure, preoperative evaluation, surgical techniques, and outcomes of revision ACLR and additional surgeries.

## 2. Methods

### 2.1. Ethnics

The use of patient data was approved by the Ethics Committee of Dalian University Affiliated Xinhua Hospital (approval number: 2023-58-01).

### 2.2. Literature search

For this narrative review, a literature search was conducted using METSTR (https://www.metstr.com/) from its inception to November 2024. The search terms used in this review are listed in the Supplementary File 1, Supplemental Digital Content, https://links.lww.com/MD/P26. A total of 85 articles were cited based on their relevance and timeliness.

## 3. Etiology of ACLR failure

Although primary ACLR failure is unmodifiable, understanding its etiology helps prevent the failure of revision ACLR. The causes of graft failure can be roughly classified as medical and nonmedical factors.

Medical factors usually refer to nonanatomic intra-articular aperture positions, also called bone tunnel positions. Anterior and distal femoral tunnels are often drilled and are associated with a higher failure rate.^[3]^ Anterior and distal femoral tunnels and posterior tibial tunnels are associated with more anterior tibial translation and internal tibial rotation.^[4]^ Allografts are associated with a higher failure rate than autografts.^[5,6]^ Hamstring tendon (HT) autografts are associated with a higher failure rate than bone-patellar tendon-bone (BPTB) autografts, while quadriceps tendon (QT) autografts show a similar failure rate to HT or BPTB autografts.^[5,7–9]^ Smaller diameter grafts and septic arthritis after primary ACLR are associated with a higher failure rate.^[5,10,11]^

Nonmedical factors usually refer to patient-related factors. The ACL is susceptible to injury at the time of anterior tibial translation, knee varus, internal rotation, and hyperextension. Because of the posterior tibial slope (PTS), proximal tibial translation can cause anterior tibial translation. Studies found that excessive medial PTS is associated with a higher failure rate.^[12,13]^ However, another study found that although the ipsilateral PTS is greater than the contralateral PTS, it is not associated with a higher failure rate.^[14]^ Additionally, medial collateral ligament injuries at the time of primary ACLR, preoperative and postoperative knee laxity, return to a high activity level, and young age are associated with a higher failure rate.^[5,15–18]^

## 4. Preoperative evaluation

Knowledge of images, surgical reports, rehabilitation, and return to sport (RTS) of primary ACLR helps evaluate the injury mechanism, surgical techniques, available autografts, and activity level. Patients’ willingness for physical activity is important because revision ACLR may not be necessary for those not demanding a high activity level.

Physical examination should include a comparison between the limbs. The acute ACL-injured leg is associated with a smaller angle of knee flexion and hip extension during stance than the uninjured leg, while the chronic ACL-injured leg shows no difference.^[19]^ Tenderness and effusion of the knee indicate that there may be a meniscus or articular cartilage injury. The Lachman test and pivot-shift test have high predictive value in diagnosing ACL rupture. A study found that every patient with a high-grade pivot shift had an anterolateral structure injury identified by the lateral exploration.^[20]^ If the patient cannot achieve full active or passive extension, prehabilitation is recommended to restore it and reduce the rate of postoperative stiffness and arthrofibrosis.^[21]^

**Figure 1.**
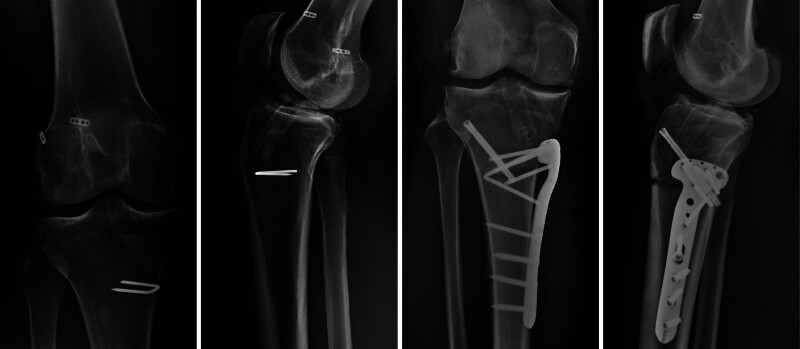
Preoperative and postoperative anteroposterior and lateral radiographs. The images are of the right knee of a 36-year-old male patient undergoing second revision anterior cruciate ligament reconstruction and anterior closing wedge high tibial osteotomy. The preoperative radiographs show 2 femoral tunnels, 2 tibial tunnels, osteophytes, medial joint space narrowing, and 2 excessive posterior tibial slopes.

**Figure 2.**
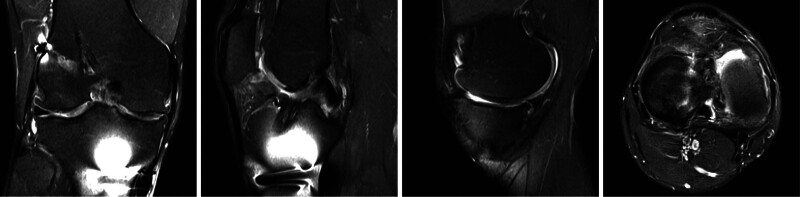
Preoperative coronal, sagittal, and axial magnetic resonance imaging images. The images are of the right knee of a 36-year-old male patient undergoing second revision anterior cruciate ligament reconstruction. The images show anterior cruciate ligament graft injury, medial meniscus injury, and medial articular cartilage injury.

**Figure 3.**
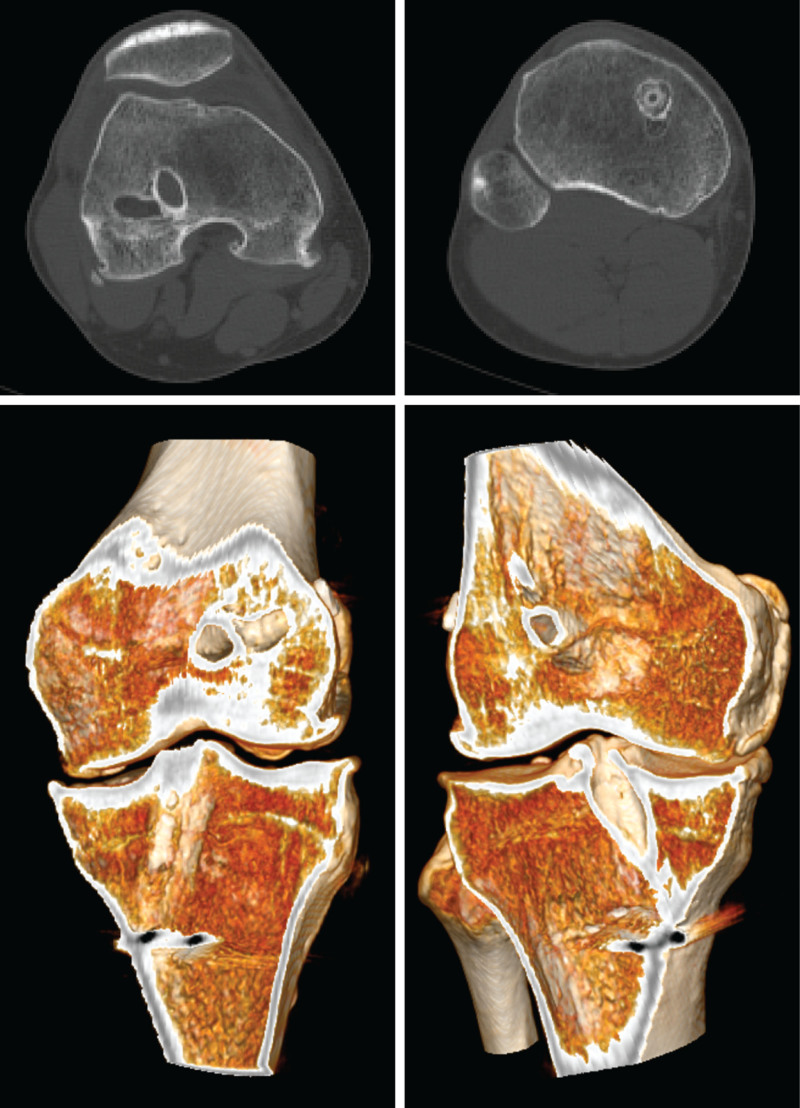
Preoperative computerized tomography axial sections and 3-dimensional reconstruction. The images are of the right knee of a 36-year-old male patient undergoing second revision anterior cruciate ligament reconstruction. The images show 2 femoral tunnels, 2 tibial tunnels, osteophytes, and medial joint space narrowing.

An anteroposterior radiograph and a lateral radiograph can be obtained for the routine evaluation of the position and size of the tunnels and hardware (Fig. 1). A full-length anteroposterior radiograph can be used to evaluate varum and valgus. A full-length lateral radiograph can be used to measure PTS.^[22]^ Magnetic resonance imaging is commonly used to evaluate soft tissue but shows no advantage in evaluating tunnels (Fig. 2).^[23,24]^ However, the precision of clinical magnetic resonance imaging limits the identification of anterolateral structure injury. Computed tomography and its 3-dimensional reconstruction provide the most comprehensive information about the position and size of the tunnels (Fig. 3).^[24]^

## 5. Surgical techniques

### 5.1. Classic tunnel management techniques

The divergent drilling technique is useful for creating a new tunnel in a different orientation from the previous one. It also helps maintain a sufficient bone bridge between the 2 tunnels, ensuring stable graft fixation. However, the smaller the sharp angle between the intratunnel graft and the intra-articular graft, the greater the force on the graft at the intra-articular aperture.

When drilling femoral tunnels, the inside-out technique includes the transtibial (TT) and anteromedial (AM) portal techniques, also called the AM or transportal technique. The TT technique is simple. However, the original TT technique usually causes an anterior femoral intra-articular aperture because the femoral tunnel is constrained by the tibial tunnel. A modified TT technique uses a more medial and proximal tibial extra-articular aperture to achieve a more anatomic femoral intra-articular aperture. However, it causes a shorter tibial tunnel and a wider tibial extra-articular aperture. Another modified TT technique uses knee varus and anterior translation and external rotation of tibia to achieve a more anatomic intra-articular aperture. The AM technique is suited for achieving anatomic intra-articular aperture. However, the AM technique causes a short tunnel and a risk of medial femoral condyle articular cartilage injury. The outside-in (OI) technique is effective in changing the tunnel trajectory as divergent drilling technique and therefore achieving adequate graft fixation because almost the entire lateral femoral condyle can be drilled. The OI technique can achieve a long tunnel, avoid posterior wall blowout. However, the OI technique requires an outer incision.^[25]^ The failure rates of the TT, AM, and OI techniques are similar after revision ACLR. However, the TT technique is associated with better clinical outcomes compared with the AM technique, while the AM technique is associated with better clinical outcomes compared with the OI technique.^[26]^

**Figure 4.**
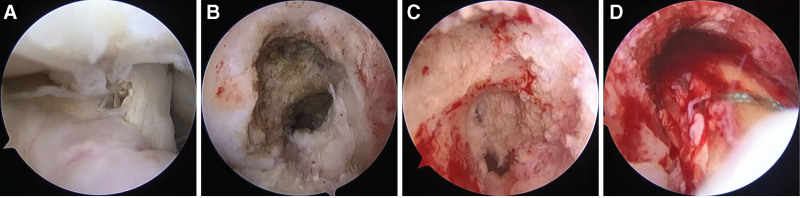
Arthroscopic images from the anterolateral portal using a 30° arthroscope. The images are of the right knee of a 36-year-old male patient undergoing second revision anterior cruciate ligament reconstruction and medial meniscus reconstruction. (A) Anterior cruciate ligament graft injury and osteophytes. (B) Previous femoral tunnel. (C) Prepared intercondylar notch for anterior cruciate ligament reconstruction using over-the-top technique. (D) Anterior cruciate ligament reconstruction using peroneus longus tendon autograft and over-the-top technique.

The over-the-top (OTT) technique avoids the challenges associated with drilling anatomic femoral tunnels and the complications caused by drilling nonanatomic femoral tunnels (Fig. 4). Because the OTT technique avoids damaging the femoral epiphysis, it is more suited for skeletally immature individuals than for skeletally mature individuals. However, the OTT technique is nonanatomic and its graft has limited effectiveness in restricting knee internal rotation in the general range of motion. Additionally, the OTT technique requires a long graft and an outer incision. The clinical outcomes of single- and double-bundle OTT reconstructions are similar to those of single- and double-bundle bone tunnel reconstructions in revision ACLR.^[27]^ Similarly, the OTT technique has a similar failure and complication rate after multiple revision ACLR compared with the AM portal technique.^[28]^

### 5.2. Staging

The 1-stage revision ACLR is indicated for mild tunnel widening (<15 mm or <150%) with no or mild overlap between the previous and new tunnels (<50% or <5 mm). An anatomic tunnel can be created by reusing the previous anatomic tunnel or by drilling a new one.

The 2-stage revision ACLR is indicated for critical tunnel widening (≥15 mm or ≥150%) and overlap (≥50% or ≥5 mm). The 2-stage revision can also be considered for other critical tunnel compromises such as insufficient bone stock and excessive hardware interference.^[29]^

Compared with the 1-stage revision, the 2-stage revision allows time for bone healing, enabling anatomic tunnels to be created. However, because of the longer rehabilitation time, the 2-stage revision may be associated with fewer health benefits from physical activities. Additionally, because of the increased number of surgeries, the 2-stage revision may be associated with higher medical costs.

### 5.3. Current 1-stage techniques for critical tunnel widening or overlap

Although critical tunnel widening or overlap is usually considered an indication for 2-stage revision,^[30,31]^ there is no universal standard to determine the stage based on the extent of tunnel widening or overlap.^[29]^ Although its effectiveness compared with 2-stage has not been tested, efforts have been made to perform 1-stage revision in these cases (Table 1).

**Table 1:** Current single-stage techniques for critical tunnel widening or overlap.

Surgical technique	Study	Study design	No. of patients	Follow-up (yr)	Tunnel diameter (mm)	Bone graft	Graft types	Highlight
Outside-in	Charles et al^[25]^	Cohort study	409	Minimum, 2	Tibial: Mean, 12 Femoral: inside-out: Mean, 11.77 outside-in: Mean, 12.23		Hamstring tendon, bone-patellar tendon-bone, quadriceps tendon	Tunnel widening (either tunnel ≥ 12 mm) and not (both tunnels < 12 mm) showed similar outcomes
Over-the-top	Gorki et al^[27]^	Cohort study	78	Minimum, 2			Hamstring tendon	Single and double over-the-top techniques and single and double bone tunnel techniques showed similar outcomes
	Emre Anil et al^[28]^	Cohort study	101	Median, 5			Hamstring tendon, bong-patellar tendon-bone, quadriceps tendon, Allograft	Over-the-top and anteromedial portal techniques showed similar outcomes
Bone grafting for sufficiently malpositioned tunnel	Jason et al^[32]^	Case series	18	Minimum, 2	Tibial: Mean, 12.3	A cylindrical bone allograft with suture anchor in proximal end and sutures pulled posteriorly		Tunnel widening (tunnel > 12 mm) and not (tunnel < 12 mm) showed similar outcomes
Rectangular tunnel	Shinichiro et al^[33]^	Cohort study	103	Median, 2.5			Bong-patellar tendon-bone	Overlap and not (includes anatomic tunnel) showed similar outcomes
Stacked screws	Ian D. et al^[34]^					Bioabsorbable or biocomposite screws		
Bone grafting after graft placement	Joachim et al^[35]^	Case series	8	Minimum, 1	Femoral: Median, 17 Tibial: Median, 16	Custom-made and cylindrical-shaped bone allografts of 8 to 10 mm diameter	Hamstring tendon, Allograft	

The OI technique is particularly suited for performing 1-stage revision when critical tunnel overlap can be avoided using this technique. The OI technique in 1-stage revision showed similar clinical outcomes between patients with and without a femoral tunnel width ≥ 12 mm.^[25]^ The OTT technique is suited for 1-stage revision when critical femoral tunnel overlap cannot be avoided by the OI technique.

Bone grafting for 1-stage revision can be considered when a critically widened tunnel is sufficiently malpositioned to avoid overlap. A 1-stage technique involves bone grafting of the posterior tibial tunnel using an allograft bone dowel, followed by drilling a new tunnel along the edge of the allograft bone dowel. There was no difference in clinical outcomes between patients with tunnel widths > 12 mm and those without.^[32]^

The rectangular tunnel technique has been used to reduce femoral tunnel size and overlap. Although the rectangular tunnel reduces the tunnel size and thickness compared with the circular tunnel with the same contact area, it requires more preparation time. When using the tunnel with a width of 5 mm and a length of 10 mm and a BPTB graft for 1-stage revision, tunnel overlap did not affect clinical outcomes.^[33]^

The stacked screws technique uses bioabsorbable or biocomposite screws to fill malpositioned and widened bone tunnels. This technique can achieve 1-stage revision but is not suited for particularly large bone defects or cases where more than 25% of the filling screw needs to be removed during drilling.^[34]^ The stacked screws technique eliminates the need for waiting for bone incorporation. However, the healing between the graft and the screw remains unclear.

A technique for 1-stage revision uses multiple customized cylinder bone allograft plugs to fill the excessively widened femoral and tibial tunnels (87.5%–250%). In this technique, bone grafts are strategically placed around the graft and impacted to fix it in an anatomic position.^[35]^ Bone grafting after graft placement eliminates the need for drilling. Fixing the graft to the tibia after bone grafting allows for final adjustments of the graft tension. However, the uneven bone grafts alter the position and shape of graft and therefore affect its remodeling.

### 5.4. Graft options

The graft options in revision ACLR remains controversial (Table 2). The activity levels and willingness of patients, the size and position of tunnels, and the cost and availability of grafts should be considered.

**Table 2:** Grafts in revision anterior cruciate ligament reconstruction.

Graft Types	Examples	Disadvantages
Autograft	Hamstring tendon autograft, bone-patellar tendon-bone autograft, quadriceps tendon autograft, peroneus longus tendon autograft	Donor site morbidity
Allograft	Hamstring tendon allograft, bone-patellar tendon-bone allograft, anterior tibial tendon allograft	High infection rate, susceptibility to donor age and irradiation
Artificial ligament	Ligament Advanced Reinforcement System	High stiffness

Autografts include ipsilateral and contralateral HT, BPTB, and QT autografts. Autografts are associated with donor site morbidity. Many surgeons still choose an ipsilateral autograft for revision ACLR if the primary ACLR was performed using an autograft.^[24]^ A study found that HT autografts are associated with a higher revision failure rate than QT autografts.^[36]^ However, other studies found no difference in revision failure rates among HT, BPTB, and QT autografts.^[37,38]^

Allografts include HT, BPTB, and anterior tibial tendon allografts. Clinical outcomes of revision ACLR using allograft are associated with donor age and irradiation.^[39,40]^ HT and BPTB allografts show similar revision failure rates.^[38]^ Allografts are associated with a higher infection rate than autografts.^[41]^ Many studies found that allografts are associated with a higher failure rate than autografts.^[38,42,43]^ However, considering the limited availability of autografts for multiple ACLR, a study found no difference in failure rates between allografts and autografts after excluding cases of multiple revision.^[42]^ Overall, the comparability of autografts and allografts in revision is limited by the variation of graft types, donor age, sterilization techniques and the number of surgeries.^[44]^

The Ligament Advanced Reinforcement System (LARS) is a trend in revision ACLR. LARS allows a short rehabilitation time.^[45]^ However, because of its high stiffness, LARS requires achieving an isometric or near-isometric reconstruction.^[46]^ A study found that knee stability after revision using LARS is better than that after using BPTB autografts.^[47]^ Another study found that knee stability and function after revision using LARS are better than those after using anterior tibial tendon allografts.^[45]^

### 5.5. Bone grafting and graft options

Bone grafting should be performed when there is insufficient bone stock to maintain stable graft fixation and sufficiently distribute the forces on the graft and bone. Bone grafting is routinely performed in 2-stage revision but may also be considered in 1-stage revision. Debride the host bone without over-reaming. Ensure sufficient contact between the bone graft and the host bone. Fix the bone graft firmly.

Bone grafts used in revision ACLR include bone autografts and allografts, synthetic bone grafts, and biologics (Table 3). An ideal bone graft should possess osteogenic, osteoinductive, and osteoconductive properties.^[48]^

**Table 3:** Bone grafts in revision anterior cruciate ligament reconstruction.

Bone graft types	Examples	Properties of ideal bone grafts	Disadvantages
Autograft	Iliac crest autograft, proximal tibia autograft, distal femur autograft	Osteogenic, osteoinductive, osteoconductive	Donor site morbidity, long preparation time
Allograft	Bone block, bone dowel, bone chip, demineralized bone matrix	Osteogenic*, osteoinductive*, osteoconductive	High infection rate*, lack of partial mechanical stability*
Synthetic bone graft	Silicate-substituted calcium phosphate, bioabsorbable screw	Osteoconductive	Limited evidence
Biologic	Bone marrow aspirate, platelet-rich plasma, bone morphogenetic protein	Osteogenic*, osteoinductive	Lack of mechanical stability

* Possessing the property in some cases.

Bone autografts are often harvested from the iliac crest. Other positions include the proximal tibia and distal femur. Cylindrical cancellous bone plug can be harvested when drilling bone tunnels using a coring reamer. Although bone autografts possess osteogenic, osteoinductive, and osteoconductive properties, their significant donor site morbidity and preparation time cause many surgeons not to use them.^[49]^

Bone allografts, harvested from donor sites similar to those of bone autografts, are available in various forms, including bone blocks, bone dowels, bone chips, and demineralized bone matrix. Bone chips and demineralized bone matrix are favorable for completely filling tunnels and defects and therefore reduce bone loss during drilling tunnels.^[50,51]^ However, because of the lack of mechanical stability, small-sized bone grafts have a risk of migration into the joint cavity.^[50]^ Allograft bone dowels can be combined with demineralized bone matrix in revision ACLR.^[52]^ Irradiation reduces the infection rate of bone allografts, but deprives their osteogenic properties, and partial mechanical stability.^[48]^ Considering the impact of irradiation on the structure, bone allografts were prepared as corticocancellous allograft chips and sterilized using supercritical carbon dioxide.^[53]^ The filling rates of femoral and tibial tunnels in imaging 3 months after bone grafting using autologous cancellous bone in revision ACLR are similar to those using allogeneic cancellous bone.^[54]^

Synthetic bone grafts are available in various forms and are of stable quality. They lack osteogenic and osteoinductive but possess osteoconductive. Silicate-substituted calcium phosphate is considered a good alternative to bone autografts in revision ACLR. The filling rates, knee stability, and clinical outcomes are similar between the silicate-substituted calcium phosphate and iliac crest autograft groups.^[55,56]^ In the stacked screws technique, bioabsorbable or biocomposite screws are used as bone grafts to fill bone tunnels.^[34]^

Biologics include bone marrow aspirate, platelet-rich plasma, and bone morphogenetic protein. Some possess osteogenic properties. All possess osteoinductive properties but lack osteoconductive properties and mechanical stability. Bone marrow aspirate is rich in mesenchymal stem cells, while platelet-rich plasma is rich in growth factors. Biologics are used to soak bone dowels and mix with bone chips to enable the migration of stem cells and growth factors into scaffolds provided by other bone grafts to enhance osteoinductive properties in revision ACLR.^[50,57]^

## 6. Additional surgeries

### 6.1. Extra-articular augmentation

ACL injuries are often accompanied by anterolateral structure injuries, particularly Kaplan fiber (the femoral attachment of the capsule-osseous layer of the iliotibial band) injuries.^[58]^ The Kaplan fiber is the primary structure that restricts tibial internal rotation at 30° or more of knee flexion. The Kaplan fiber, anterolateral capsule, and anterolateral ligament restrict tibial internal rotation in the ACL-deficient knee.^[59]^

Because the anterolateral structure is commonly injured in high-grade pivot shift, extra-articular augmentation can be indicated for ACLR failure with a high-grade pivot shift.^[20,60]^ However, some surgeons consider extra-articular augmentation unnecessary at the time of revision ACLR.^[33]^ Considering the cooperativity between the ACL and the anterolateral structure in restricting tibial internal rotation and anterior tibial translation, extra-articular augmentation may not be necessary at the time of ACLR using a large graft. Additionally, a large or short graft may cause excessive restriction.

Studies found that adding extra-articular augmentation at the time of revision ACLR reduces pivot shift and failure rates, while no difference was found between iliotibial band tenodesis and anterolateral ligament reconstruction.^[61–64]^ However, other studies found that adding extra-articular augmentation at the time of revision ACLR does not improve clinical outcomes.^[65,66]^ The different results of adding extra-articular augmentation at the time of revision ACLR may be caused by the different severity of anterolateral complex injuries in patients selected for extra-articular augmentation among studies. Additionally, extra-articular augmentation at the time of revision ACLR can cause a higher rate and longer time of lateral pain.^[63]^

### 6.2. Osteotomy

Varus knee is associated with higher tension on the ACL graft when the tibia moves proximally.^[67]^ Varus knee is more common at the time of revision ACLR than at the time of primary ACLR.^[68]^ Medial opening wedge high tibial osteotomy (HTO) or lateral closing wedge HTO may be indicated for ACL injury, varus knee, mild to moderate medial osteoarthritis, and age < 65 years. Medial opening wedge HTO is popular because it precisely corrects alignment and only requires a single cut. However, the healing is slow and may require bone grafting.

Excessive PTS is associated with higher tension on the ACL graft when the tibia moves proximally.^[67]^ Consistent with primary ACLR, excessive PTS is associated with a higher failure rate in revision ACLR.^[69,70]^ Anterior closing wedge HTO may be indicated for ACL injury with PTS > 12° (Fig. 1).^[71–73]^ Considering the significant trauma caused by HTO, if the contralateral knee has a PTS > 12° but no history of ACL injury, anterior closing wedge HTO may not be necessary.

### 6.3. Meniscus and articular cartilage surgeries

A study found that the incidences of meniscus and articular cartilage injuries in primary ACLR were 44.6% and 7.0%, while the incidences of meniscus and articular cartilage injuries in revision ACLR observed in the same patients were 70.0% and 15.5%.^[2]^ Meniscus and articular cartilage injuries at the time of revision ACLR are associated with poor clinical outcomes.^[74,75]^ Meniscus and articular cartilage injuries should be addressed (Fig. 4). Meniscus repair and replacement at the time of revision have shown good outcomes.^[76–78]^

## 7. Outcomes

Overall, revision ACLR improves the knee function.^[79–81]^ Revision ACLR shows similar knee stability but worse knee function and a higher failure rate compared with primary ACLR.^[82]^ A possible explanation for the difference is that the incidences of meniscus and articular cartilage injuries are higher at the time of revision.^[2,75]^ Similarly, knee function after multiple revision is worse than that after single revision.^[79,80]^

Return to sport is a key criterion for evaluating revision ACLR. A study found that 39% return to the same level sport as before injury 5 years after revision, while 61% return to the lower level sport.^[83]^ Not RTS 2 years after revision is associated with current smoking, female gender, and recreational-level activity.^[84]^ The rate of return to the same level sport as before injury 1 year after revision ACLR is lower than that after primary ACLR.^[85]^ Similarly, the rate of return to the same level sport as before the injury 1 year after multiple revision is lower than that after single revision.^[79]^

## 8. Future directions

Considering that current 1-stage techniques in these cases have not been compared with 2-stage techniques, an important future direction in this field is to expand the indications for 1-stage revision in cases of critical tunnel widening or overlap based on strong evidence. Although the application of LARS in revision ACLR has accumulated evidence in recent years, more evidence is awaited. Considering that the comparability of studies on extra-articular augmentation at the time of revision ACLR is limited by the variations in the indications for extra-articular augmentation, future studies evaluating and comparing the effects of extra-articular augmentation in revision ACLR based on clear indications will be valuable. Considering that anterior closing wedge HTO in revision ACLR is not supported by some experts, the indications require further clarification.

## 9. Conclusions

Understanding the etiology of ACLR failure helps prevent the failure of revision ACLR. A thorough preoperative evaluation is important for personalized revision. There have been efforts to expand the criteria of 1-stage revision. There have been more bone graft and graft options in recent years. Extra-articular augmentation and osteotomy play an important role in revision. Meniscus and articular cartilage injuries should be addressed. Although the overall outcomes of revision ACLR are worse than those of primary ACLR, revision ACLR remains important for improving knee function and RTS.

## Author contributions

**Supervision:** Rui-Xin Li.

**Conceptualization:** Tian-Wang Zhu.

**Methodology:** Tian-Wang Zhu. 

**Investigation:** Tian-Wang Zhu. 

**Visualization:** Tian-Wang Zhu.

**Writing – original draft:** Tian-Wang Zhu.

## Supplementary Material


