# Supplementary material for: Revision anterior cruciate ligament reconstruction and additional surgeries: A review
Source: Medicine (Baltimore). 2025 May 23;104(21):e42620. doi: 10.1097/MD.0000000000042620 (PMC12113963; doi:10.1097/MD.0000000000042620)
Supplement: Supplementary file 1 [file medi-104-e42620-s001.pdf]

Search terms

(anterior cruciate ligament[TI] OR anterior cruciate ligaments[TI] OR ACL[TI]) AND (reoperation[TI] OR revision[TI] OR Surgical Revision[TI] OR Surgery, Repeat[TI] OR Revision, Surgical[TI] OR Revision Surgery[TI] OR Revision Surgeries[TI] OR Surgery, Revision[TI] OR Repeat Surgery[TI] OR Revision, Joint[TI] OR Joint Revision[TI]).

(ACLR[TI] OR anterior cruciate ligament reconstruction[TI]) AND alignment[TIAB].

(ACLR[TI] OR anterior cruciate ligament reconstruction[TI]) AND single-stage revision[TIAB].

osteotomy[TI] AND valgus malalignment[TI].

osteotomy[TI] AND varus malalignment[TI].

(anterior cruciate ligament[TI] OR anterior cruciate ligaments[TI] OR ACL[TI]) AND (reoperation[TI] OR revision[TI] OR Surgical Revision[TI] OR Surgery, Repeat[TI] OR Revision, Surgical[TI] OR Revision Surgery[TI] OR Revision Surgeries[TI] OR Surgery, Revision[TI] OR Repeat Surgery[TI] OR Revision, Joint[TI] OR Joint Revision[TI]) AND menisc*[TI].

(anterior cruciate ligmant*[TI] OR ACL*[TI]) AND concomitant[TI] AND injur*[TI].

(artificial ligament*[TI] OR LARS*[TI]) AND (anterior cruciate ligament*[TI] OR ACL*) AND revision[TI].

(ACL*[TI] OR anterior cruciate ligament*[TI]) AND revision[TI] AND (infect*[TIAB] OR inflamm*[TIAB]).

(ACL*[TI] OR anterior cruciate ligament*[TI]) AND (range of motion[TI] OR ROM[TI]).

(ACL*[TI] OR anterior cruciate ligament*[TI]) AND revision[TI] AND (complex[TI] OR collateral[TI]).

(ACL*[TI] OR anterior cruciate ligament*[TI]) AND revision[TI] AND menisc*[TI].

((anterior cruciate ligament* revision[TI] OR revision anterior cruciate ligament*[TI] OR ACL* revision[TI] OR revision ACL*[TI]) NOT revision rate[TI]) AND graft*[TI] NOT bone*[TI].

((anterior cruciate ligament* revision[TI] OR revision anterior cruciate ligament*[TI]

OR ACL* revision[TI] OR revision ACL*[TI]) NOT revision rate[TI]) AND bone[TI].

((anterior cruciate ligament* revision[TI] OR revision anterior cruciate ligament*[TI] OR ACL* revision[TI] OR revision ACL*[TI]) NOT revision rate[TI]) AND tunnel*[TI].

((anterior cruciate ligament* revision[TI] OR revision anterior cruciate ligament*[TI] OR ACL* revision[TI] OR revision ACL*[TI]) NOT revision rate[TI]) AND screw*[TI].

over the top[TI] AND (patellar[TI] OR quadricep*[TI]).

((anterior cruciate ligament* revision[TI] OR revision anterior cruciate ligament*[TI] OR ACL* revision[TI] OR revision ACL*[TI]) NOT revision rate[TI]) AND outcome*[TI].

((anterior cruciate ligament* revision[TI] OR revision anterior cruciate ligament*[TI] OR ACL* revision[TI] OR revision ACL*[TI]) NOT revision rate[TI]) AND osteotom*[TI].

osteotom*[TI] AND tibial[TI] AND deflexion[TI]. osteotom*[TI] AND tibial[TI] AND slope[TI] AND reduc*[TI].

iliotibial tract*[TI] AND biomechanic*[TI]. anterolateral*[TI] AND biomechanic*[TI].

((anterior cruciate ligament* revision[TI] OR revision anterior cruciate ligament*[TI] OR ACL* revision[TI] OR revision ACL*[TI]) NOT revision rate[TI]) AND anterolateral*[TI] OR (ALL[TI] OR extraarticular[TI] OR extra-articular[TI] OR LET[TI]).

((anterior cruciate ligament* revision[TI] OR revision anterior cruciate ligament*[TI] OR ACL* revision[TI] OR revision ACL*[TI]) NOT revision rate[TI]) AND menisc*[TI].

((anterior cruciate ligament* revision[TI] OR revision anterior cruciate ligament*[TI] OR ACL* revision[TI] OR revision ACL*[TI]) NOT revision rate[TI]) AND (chondr*[TI] OR cartilage*[TI]).

((anterior cruciate ligament* revision[TI] OR revision anterior cruciate ligament*[TI] OR ACL* revision[TI] OR revision ACL*[TI]) NOT revision rate[TI]) AND (primary[TI] OR index[TI]) AND (sport*[TI] OR RTS*[TI]).

((anterior cruciate ligament* revision[TI] OR revision anterior cruciate ligament*[TI]

OR ACL* revision[TI] OR revision ACL*[TI]) NOT revision rate[TI]) AND (radiograph*[TI] OR X-ray[TI] OR MRI[TI] OR resonance*[TI] OR CT[TI] OR tomograph*[TI]).

((anterior cruciate ligament* revision[TI] OR revision anterior cruciate ligament*[TI] OR ACL* revision[TI] OR revision ACL*[TI]) NOT revision rate[TI]) AND gait*[TI].

((anterior cruciate ligament* revision[TI] OR revision anterior cruciate ligament*[TI] OR ACL* revision[TI] OR revision ACL*[TI]) NOT revision rate[TI]) AND KT*[TI].

((anterior cruciate ligament* revision[TI] OR revision anterior cruciate ligament*[TI] OR ACL* revision[TI] OR revision ACL*[TI]) NOT revision rate[TI]) AND (OTT*[TI] OR over the top[TI]).

((anterior cruciate ligament* revision[TI] OR revision anterior cruciate ligament*[TI] OR ACL* revision[TI] OR revision ACL*[TI]) NOT revision rate[TI]) AND stage*[TI].

(anterior cruciate ligament*[TI] OR ACL*[TI]) AND (position[TI] OR placement[TI]) AND (biomechanical[TI] OR mechanic*[TI] OR stabilit*[TI] OR translation*[TI]).

(anterior cruciate ligament* revision[TI] OR revision anterior cruciate ligament*[TI] OR ACL* revision[TI] OR revision ACL*[TI]) AND (rate[TI] OR risk[TI]).

(anterior cruciate ligament* revision[TI] OR revision anterior cruciate ligament*[TI] OR ACL* revision[TI] OR revision ACL*[TI]) AND (one-stage[TI] OR 1-stage[TI]) AND (two-stage[TI] OR 2-stage[TI]).

(anterior cruciate ligament* revision[TI] OR revision anterior cruciate ligament*[TI] OR ACL* revision[TI] OR revision ACL*[TI]) NOT revision rate[TIAB] AND osteoarthritis[TIAB] NOT osteoarthritis outcome[TIAB]. (anterior cruciate ligament* revision[TI] OR revision anterior cruciate ligament*[TI] OR ACL* revision[TI] OR revision ACL*[TI]) AND infection[TI].

(caus*[TI] OR etiolog*[TI]) AND tunnel[TI] AND (dilation[TI] OR widen*[TI] OR enlarg*[TI]).
